# Multidisciplinary team care and outcomes in FNCLCC grade 3 soft tissue sarcoma: a propensity score–matched and weighted cohort study

**DOI:** 10.3389/fonc.2026.1827237

**Published:** 2026-06-10

**Authors:** Zhangfu Li, Xinyu Li, Baicheng Yang, Xiaoyang Li, Luqiang Wang, Ting Liu, Xinxin Zhang, Zhenguo Zhao, Libin Xu, Helin Feng, Zhenfeng Li, Shengji Yu

**Affiliations:** Department of Orthopaedics, National Cancer Center/National Clinical Research Center for Cancer/Cancer Hospital, Chinese Academy of Medical Sciences and Peking Union Medical College, Beijing, China

**Keywords:** distant metastasis, FNCLCC grade 3, local recurrence, multidisciplinary team, soft tissue sarcoma

## Abstract

**Background:**

Multidisciplinary team (MDT) care is widely advocated for soft tissue sarcoma (STS), yet real-world evidence in high-grade disease remains limited and subject to confounding. We investigated whether pre-treatment MDT care was associated with improved outcomes in FNCLCC grade 3 STS.

**Methods:**

This retrospective cohort study included consecutive adults with pathologically confirmed FNCLCC grade 3 STS treated at the Department of Orthopaedics, Cancer Hospital, Chinese Academy of Medical Sciences (2010–2022). MDT care was defined as formal discussion after pathological diagnosis and before definitive treatment. Primary endpoints were 3-year local recurrence and distant metastasis from diagnosis (administrative censoring at 36 months). Propensity scores were estimated using logistic regression; confounding was addressed using 1:1 propensity score matching (PSM) and stabilized inverse probability of treatment weighting (IPTW). Odds ratios (ORs) and Kaplan–Meier analyses were applied.

**Results:**

Among 338 patients (MDT, n=88; non-MDT, n=250), MDT care was associated with lower 3-year local recurrence (6.8% vs 21.2%; unadjusted OR = 0.272, P = 0.002), with consistent results after PSM (paired OR = 0.259, P = 0.021) and IPTW (OR = 0.296, P = 0.008). By contrast, 3-year distant metastasis did not differ significantly between groups in unadjusted, matched, or weighted analyses (weighted OR = 0.846, 95% CI 0.418–1.714; P = 0.643), although the confidence interval did not exclude a potentially moderate clinically meaningful benefit.

**Conclusions:**

In FNCLCC grade 3 STS, pre-treatment MDT care was associated with improved 3-year local control but not with a significant reduction in 3-year distant metastasis.

## 1 Introduction

Soft tissue sarcomas (STS) are a rare and biologically heterogeneous group of mesenchymal malignancies, accounting for approximately 1% of adult cancers and comprising more than 100 histologic subtypes with diverse clinical behaviors (1, 2). This rarity and heterogeneity make accurate diagnosis and risk stratification essential, as treatment decisions often require coordination across multiple specialties (3).

Histologic grade is among the most important prognostic determinants in STS. The French Fédération Nationale des Centres de Lutte Contre le Cancer (FNCLCC) grading system was developed to provide reproducible prognostic stratification and has been shown to correlate strongly with the risk of metastasis and survival (4, 5). In clinical practice, high-grade STS—particularly FNCLCC grade 3—represents a subgroup with substantial risks of both local recurrence and distant metastasis, where early management decisions may have outsized downstream consequences (6).

Curative-intent management of localized STS typically relies on precise pretreatment evaluation (appropriate imaging and biopsy), oncologically sound surgical planning, and selective integration of radiotherapy and systemic therapy based on histology and risk (7). Contemporary clinical practice guidelines emphasize that care should be delivered by experienced multidisciplinary teams in specialized centers, reflecting the complexity of diagnosis (including expert pathology review), staging, biopsy technique, surgical margin planning, and multimodality sequencing (8, 9).

Beyond guideline statements, observational evidence increasingly suggests that centralization of sarcoma care and adherence to clinical practice guidelines are associated with improved outcomes. In a prospective, population-based cohort, treatment within expert centers and adherence to guideline-concordant initial management were linked to better survival for patients with localized sarcoma (10). Nevertheless, real-world implementation of multidisciplinary team (MDT) care is non-random, and patients discussed at MDT meetings may systematically differ from those managed outside MDT pathways, highlighting the need for analytic strategies that address confounding.

Unplanned excision (“whoops” surgery) exemplifies this challenge. Unplanned resections of high-grade STS are common in referral settings and have been associated with high rates of residual tumor and substantially increased local recurrence compared with planned oncologic resection, potentially complicating subsequent management (11). Local recurrence itself is clinically meaningful, as it may signal aggressive tumor biology and is associated with subsequent metastatic progression and tumor-related mortality in extremity STS cohorts (12, 13).

Given these considerations, evaluating MDT care within a high-risk and clinically consequential subgroup may provide clearer insight into its potential benefits. Therefore, we conducted a retrospective cohort study focused on FNCLCC grade 3 STS to compare outcomes between MDT and non-MDT care. Using propensity score methods, we aimed to estimate the association of MDT care with 3-year local recurrence and 3-year distant metastasis, and to characterize time-to-event outcomes using Kaplan–Meier analyses.

## 2 Methods

### 2.1 Study design and setting

This retrospective cohort study was conducted at the Department of Orthopaedics, Cancer Hospital, Chinese Academy of Medical Sciences. Consecutive patients treated between January 2010 and December 2022 were screened.

### 2.2 Study population

**Figure 1:**
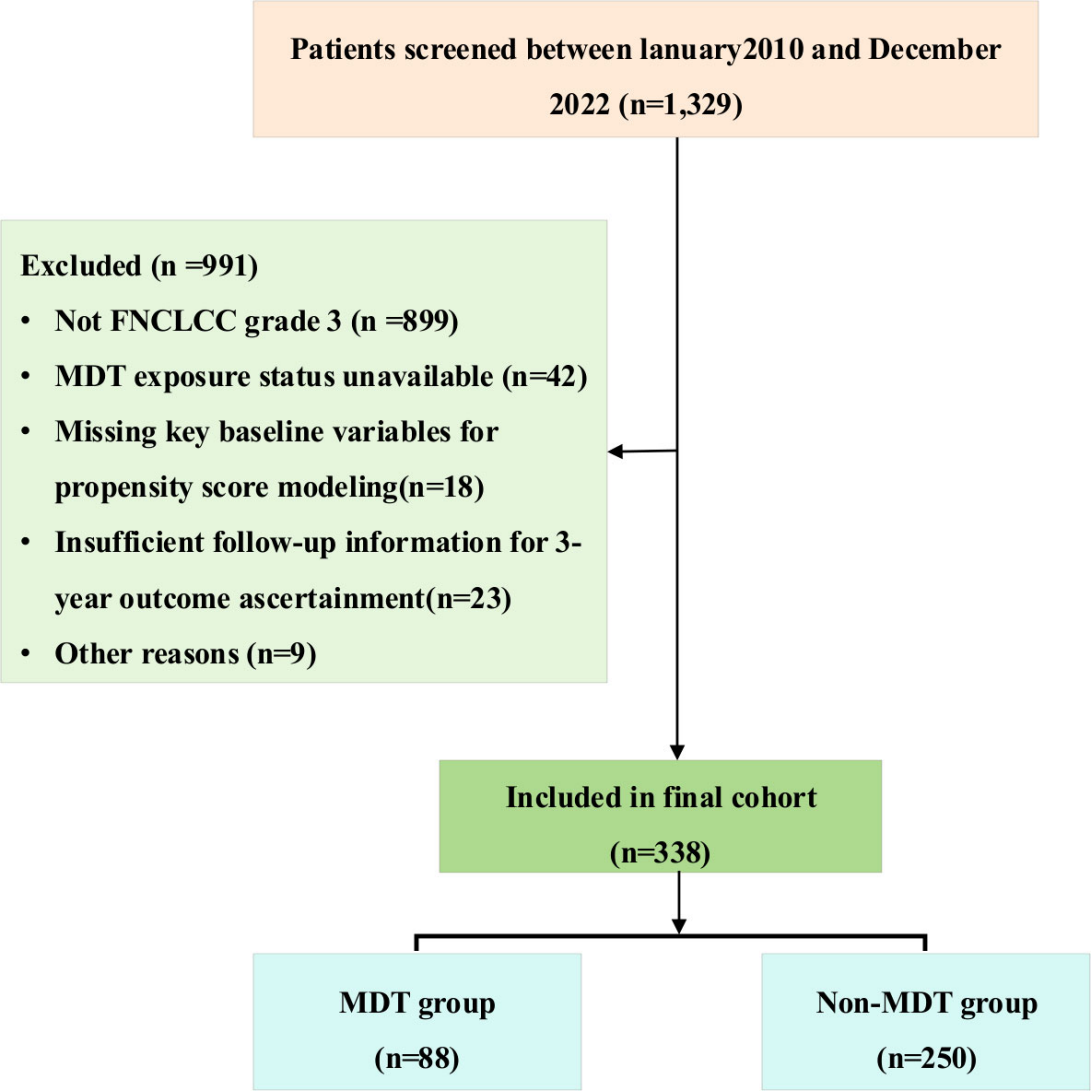
Flow diagram of patient selection.

Eligible patients were adults with pathologically confirmed soft tissue sarcoma graded as FNCLCC grade 3 who received curative-intent management at our institution. Of 1,329 patients initially screened, 899 were excluded because they were not FNCLCC grade 3, 42 because MDT exposure status could not be ascertained, 18 because key baseline variables required for propensity score modeling were unavailable, 23 because follow-up information was insufficient for 3-year outcome ascertainment, and 9 for other reasons. The final analytic cohort comprised 338 patients. A flow diagram of patient selection is shown in **Figure 1**.

### 2.3 Exposure definition: MDT care

The exposure of interest was pre-treatment multidisciplinary team (MDT) care. MDT care was defined as formal discussion at a sarcoma MDT meeting after confirmation of diagnosis and before initiation of definitive treatment, with documented recommendations recorded by a designated coordinator. The MDT meeting consisted of at least four orthopedic oncology surgeons, two medical oncologists, two pathologists, one radiation oncologist, and one radiologist; all participants were at or above the level of associate chief physician. Patients were classified into the MDT group if the index case was discussed during this interval; otherwise, patients were classified as non-MDT. Although MDT recommendations were documented, adherence to individual recommendations was not systematically captured in the study database and therefore was not assessed in the present analysis.

Definitive treatment initiation was defined as the first curative-intent intervention for the index tumor (surgery and/or radiotherapy and/or systemic therapy, whichever occurred first).

### 2.4 Data collection and covariates

Data were abstracted from electronic medical records and pathology reports using a standardized form. Covariates were selected *a priori* based on clinical relevance and potential association with MDT allocation and outcomes, including age, sex, BMI, ECOG performance status, Charlson comorbidity index, smoking status, tumor site, tumor depth, tumor size (continuous) and size category (≤5 cm, 5–10 cm, >10 cm), histology subtype, presentation status (primary vs recurrent), prior unplanned excision (“whoops”), neurovascular involvement, and bone involvement. Detailed data on surgical margin width (mm), radiotherapy dose/fractionation, chemotherapy regimen, and time from pathological diagnosis to treatment initiation were not systematically captured in a sufficiently complete and standardized manner in the retrospective study database and therefore were not included in the present analysis.

### 2.5 Outcomes and follow-up

Time zero was defined as the date of pathological diagnosis (the date of first pathological confirmation of the index tumor). The primary outcomes were 3-year local recurrence and 3-year distant metastasis. Local recurrence was defined as radiologic and/or pathologic evidence of tumor recurrence at or adjacent to the primary site after definitive management. Distant metastasis was defined as radiologic and/or pathologic evidence of metastatic disease outside the primary region. Outcomes were ascertained from follow-up clinic records and telephone follow-up documentation.

For binary 3-year endpoints, events were counted if they occurred within 36 months after pathological diagnosis; patients without events were administratively censored at 36 months.

Time-to-event outcomes included local recurrence-free survival (LRFS) and distant metastasis-free survival (DMFS), defined as time from pathological diagnosis to the first occurrence of local recurrence or distant metastasis, respectively, with administrative censoring at 36 months for 3-year Kaplan–Meier curves.

For the primary analyses, the study endpoints were prespecified as fixed 3-year outcomes (3-year local recurrence and 3-year distant metastasis), and patients without sufficient information for 3-year outcome ascertainment were excluded accordingly. Kaplan–Meier curves were provided as complementary descriptive displays of event timing within the 36-month horizon.

### 2.6 Statistical analysis

Continuous variables are presented as median (interquartile range) and compared using the Mann–Whitney U test. Categorical variables are presented as counts (percentages) and compared using the chi-square test or Fisher’s exact test, as appropriate. Covariate balance between groups was assessed using standardized mean differences (SMD).

To reduce confounding, propensity scores representing the probability of receiving MDT care were estimated using logistic regression including age, sex, BMI, ECOG performance status, Charlson comorbidity index, smoking status, tumor site, tumor depth, tumor size category, histology subtype, presentation status, prior unplanned excision (“whoops”), neurovascular involvement, and bone involvement. Two complementary approaches were applied: propensity score matching (PSM) and inverse probability of treatment weighting (IPTW). For PSM, 1:1 nearest-neighbor matching without replacement was performed within a caliper of 0.2 standard deviations of the logit of the propensity score. Covariate balance after matching was assessed using absolute standardized mean differences (SMDs), with an absolute SMD < 0.1 considered indicative of acceptable balance. For IPTW, stabilized weights were used to construct a weighted pseudo-population with improved covariate balance and were calculated as P(T = 1)/PS for MDT patients and P(T = 0)/(1−PS) for non-MDT patients, where P(T = 1) and P(T = 0) represent the marginal probabilities of treatment assignment. No weight trimming was applied. Weight distribution and covariate balance after weighting were further assessed using a histogram of stabilized weights, effective sample size (ESS), and a Love plot of standardized mean differences before and after IPTW.

For binary 3-year outcomes, effect estimates were expressed as odds ratios (ORs). Unadjusted comparisons used Fisher’s exact test. In the matched cohort, paired comparisons used McNemar’s exact test. In weighted analyses, ORs and 95% confidence intervals (CIs) were derived from weighted logistic regression. Kaplan–Meier methods were used to estimate LRFS and DMFS as complementary descriptive time-to-event summaries, whereas the primary inferential analyses were based on prespecified fixed 3-year binary outcomes.

All analyses were performed using R (version 4.4.3). Two-sided P values <0.05 were considered statistically significant.

## 3 Results

### 3.1 Study population

A total of 338 patients with FNCLCC grade 3 soft tissue sarcoma were included (88 in the MDT group and 250 in the non-MDT group). Within 3 years, local recurrence occurred in 59 patients (17.5%), and distant metastasis occurred in 54 patients (16.0%).

### 3.2 Baseline characteristics

Baseline characteristics are summarized in **Table 1**. Overall, the MDT and non-MDT groups were broadly comparable in key demographic and tumor-related variables. Notably, the MDT group tended to include patients with a larger tumor size distribution and a higher proportion of ECOG performance status ≥2, while modest imbalances were also observed for presentation status and prior unplanned excision. To demonstrate covariate balance after matching, **Table 1** also reports post-match SMDs calculated in the 75 matched pairs, and **Supplementary Figure 3** provides a graphical summary of covariate balance before and after PSM.

**Table 1:** Baseline characteristics of patients with FNCLCC grade 3 soft tissue sarcoma.

Characteristic	Non-MDT (n=250)	MDT (n=88)	P value	SMD before PSM	SMD after PSM
Patient characteristics					
Age, years	50.0 (39.0–61.0)	52.5 (31.2–63.0)	0.535	0.033	0.081
BMI, kg/m²	24.5 (22.4–26.2)	23.9 (21.7–25.9)	0.117	0.185	0.060
ECOG PS (≥2)	12 (4.8%)	15 (17.0%)	0.001	0.393	0.086
Charlson comorbidity index	0.0 (0.0–1.0)	0.0 (0.0–1.0)	0.301	0.086	0.043
Smoking status			0.512	0.163	0.204
Never	134 (53.6%)	40 (45.5%)			
Former	62 (24.8%)	23 (26.1%)			
Current	46 (18.4%)	22 (25.0%)			
Unknown	8 (3.2%)	3 (3.4%)			
Tumor characteristics					
Tumor site			0.351	0.183	0.038
Upper limb	48 (19.2%)	11 (12.5%)			
Lower limb	120 (48.0%)	47 (53.4%)			
Trunk	82 (32.8%)	30 (34.1%)			
Tumor depth (deep)	210 (84.0%)	71 (80.7%)	0.509	0.087	0.097
Tumor size, cm	5.0 (3.0–7.0)	5.5 (4.0–10.0)	0.033	0.396	0.095
Tumor size category			<0.001	0.430	0.098
≤5 cm	146 (58.4%)	43 (48.9%)			
5–10 cm	91 (36.4%)	28 (31.8%)			
>10 cm	13 (5.2%)	17 (19.3%)			
Histology subtype (overall)			0.472	0.202	0.165
UPS	67 (26.8%)	18 (20.5%)			
LMS	31 (12.4%)	14 (15.9%)			
DDLPS	32 (12.8%)	7 (8.0%)			
SS	26 (10.4%)	9 (10.2%)			
MPNST	17 (6.8%)	8 (9.1%)			
MFS	23 (9.2%)	14 (15.9%)			
FS	25 (10.0%)	10 (11.4%)			
Other	29 (11.6%)	8 (9.1%)			
Presentation status (recurrent)	49 (19.6%)	10 (11.4%)	0.102	0.228	0.332
Prior unplanned excision (whoops)	61 (24.4%)	14 (15.9%)	0.104	0.212	0.000
Neurovascular involvement	58 (23.2%)	23 (26.1%)	0.565	0.068	0.123
Bone involvement	20 (8.0%)	8 (9.1%)	0.822	0.039	0.082
Follow-up					
Follow-up, months	45.0 (39.9–49.6)	45.5 (39.2–49.7)	0.807	0.027	0.087

Values are presented as median (interquartile range) or n (%). SMD, standardized mean difference; PSM, propensity score matching; MDT, multidisciplinary team; ECOG PS, Eastern Cooperative Oncology Group performance status. Post-match SMDs were calculated in the 75 matched pairs. For categorical variables with more than two levels, the largest absolute SMD across levels is reported.

### 3.3 Summary of effect estimates

**Figure 2:**
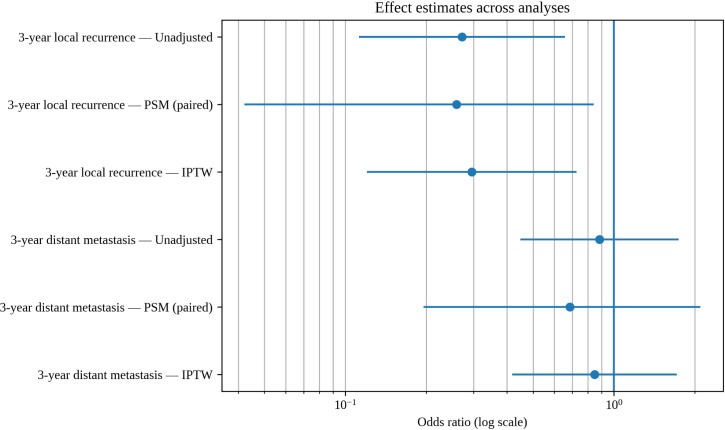
Forest plot of effect estimates for 3-year local recurrence and distant metastasis.

**Figure 2** summarizes effect estimates across unadjusted, PSM, and IPTW analyses. The association was consistently in favor of MDT care for 3-year local recurrence, whereas estimates for distant metastasis were weaker and more uncertain. After IPTW, the weighted sample sizes were 251.8 in the non-MDT group and 85.1 in the MDT group, with corresponding effective sample sizes of 220.4 and 66.6, respectively (overall ESS: 286.3). **Supplementary Figure 1** shows covariate balance before and after IPTW, and **Supplementary Figure 2** shows the distribution of stabilized weights.

### 3.4 Local recurrence

**Figure 3:**
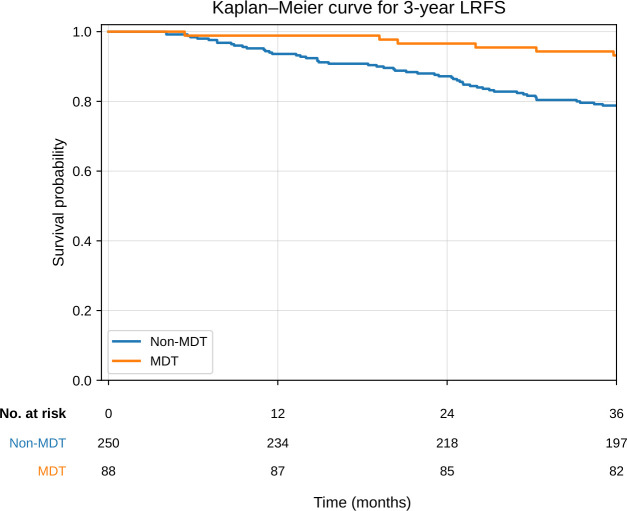
Kaplan–Meier curve for 3-year LRFS.

In the unadjusted analysis, the MDT group had a lower 3-year local recurrence rate than the non-MDT group (6.8% vs 21.2%; OR = 0.272, P = 0.002; **Table 2**). Findings were directionally consistent across sensitivity analyses, and the 3-year LRFS curves are shown in **Figure 3**.

**Table 2:** Three-year local recurrence and distant metastasis outcomes across analytic approaches.

Analysis	3-year local recurrence			3-year distant metastasis			
	non-MDT	MDT	Effect	non-MDT	MDT	Effect	P
Unadjusted	53/250 (21.2%)	6/88 (6.8%)	OR=0.272	41/250 (16.4%)	13/88 (14.8%)	OR=0.884	0.866
PSM (75 pairs)	15/75 (20.0%)	5/75 (6.7%)	Paired OR = 0.259	12/75 (16.0%)	9/75 (12.0%)	Paired OR = 0.684	0.607
IPTW	52.8/258.6 (20.4%)	5.8/82.0 (7.0%)	OR=0.296 (0.121–0.727)	41.8/258.6 (16.1%)	11.5/82.0 (14.0%)	OR=0.846 (0.418–1.714)	0.643

Unadjusted and PSM cells are shown as n/N (%). IPTW cells are shown as weighted events/weighted N (%). Unadjusted P values are from Fisher’s exact test; PSM results are paired estimates (McNemar exact); IPTW results are from weighted logistic regression.

### 3.5 Distant metastasis

**Figure 4:**
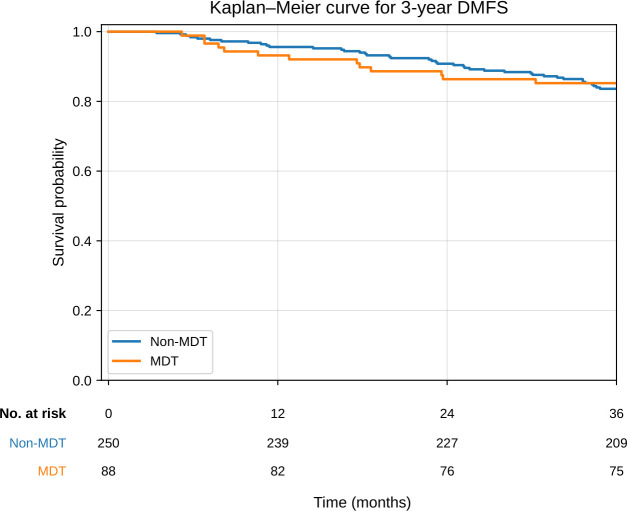
Kaplan–Meier curve for 3-year DMFS.

For 3-year distant metastasis, event rates were 13/88 (14.8%) in the MDT group and 41/250 (16.4%) in the non-MDT group (**Table 2**), corresponding to an absolute risk difference of −1.6% (95% CI, −10.4% to 7.1%) and an unadjusted OR of 0.884 (P = 0.866). Although this difference was not statistically significant, the confidence interval was wide, and the estimates from matched and weighted analyses remained directionally similar. The 3-year DMFS curves with censoring ticks and numbers at risk are shown in **Figure 4**.

## 4 Discussion

In this retrospective cohort of patients with FNCLCC grade 3 soft tissue sarcoma treated between 2010 and 2022, we evaluated whether pre-treatment MDT care was associated with improved oncologic outcomes compared with non-MDT management. Using complementary propensity score approaches (matching and inverse probability weighting), we found that MDT care was consistently associated with a lower risk of 3-year local recurrence, with concordant separation of the LRFS curves. In contrast, the association between MDT care and 3-year distant metastasis was modest and not statistically significant across analytic strategies, with largely overlapping DMFS curves. Together, these findings suggest that MDT care was more strongly associated with a lower risk of 3-year local recurrence than with 3-year distant metastasis in high-grade STS, whereas short-term metastatic outcomes appeared less sensitive to MDT-based management within the 3-year horizon.

Several mechanisms may explain why MDT care was associated with improved local control in this high-grade cohort. In a nationwide sarcoma network, presentation to a specialized multidisciplinary tumor board before treatment was linked to better compliance with key quality steps (e.g., biopsy before surgery, appropriate imaging, and higher quality initial surgery) and was associated with improved local relapse-free outcomes, supporting the hypothesis that MDT input may be associated with better local disease management (14). In addition, local control in STS depends on coordinated delivery of surgery and radiotherapy; an ASTRO clinical practice guideline provides evidence-based recommendations on indications, sequencing, and planning of radiotherapy for operable adult STS, reinforcing the role of multidisciplinary decision-making in optimizing local therapy (15). Finally, “whoops” pathways highlight how MDT input may matter most in complex referrals: unplanned excision is often performed without appropriate radiologic evaluation and is frequently followed by residual tumor at re-excision, illustrating the need for coordinated re-resection planning and multimodality integration to reduce avoidable local failure (16).

In contrast to local recurrence, we did not observe a statistically significant association between MDT care and 3-year distant metastasis. This pattern is biologically plausible because metastatic dissemination in high-grade STS is driven predominantly by intrinsic tumor aggressiveness and only partially modifiable clinical factors, which MDT care may not fully overcome within a 3-year horizon. In a population-based cohort of extremity and trunk wall STS that specifically evaluated patients who were event-free at 3 years, malignancy grade and marginal surgical margins remained predictors of late metastasis, highlighting that metastatic risk can persist beyond early follow-up (17). Consistently, a contemporary cohort study identified tumor size and histologic high grade as major predictors of distant and lung metastasis, underscoring the dominant role of tumor biology (18). Moreover, the benefit of perioperative systemic therapy for preventing distant relapse in localized STS remains heterogeneous and uncertain; adjuvant chemotherapy is not a universal standard and is generally considered on an individualized basis in high-risk patients (19, 20). Given the limited number of metastatic events (n=54) and the wide confidence intervals around the effect estimates, a modest but clinically meaningful reduction in distant metastasis cannot be excluded, even though the observed association did not reach statistical significance.

Our findings are consistent with prior real-world evidence suggesting that earlier expert multidisciplinary input and specialist-center management improve the quality of initial care and, consequently, local disease control in STS. In the French NETSARC nationwide registry, surgical management within reference centers was associated with reduced risks of relapse and death, supporting the broader concept that specialist pathways deliver higher-quality local therapy at scale (21). More recently, a comprehensive cancer center analysis further showed that adherence to treatments recommended after interdisciplinary tumor board discussion was associated with improved outcomes in soft tissue sarcoma, reinforcing the notion that MDT benefits may be mediated through more consistent, guideline-concordant execution of complex multimodality plans rather than through changes in tumor biology itself (22). Taken together with our propensity score–based results, these data support an association between MDT-based management and improved local control in high-grade STS, while highlighting that additional strategies are likely required to meaningfully impact metastatic risk.

Methodologically, our analysis strengthens the inference that the observed association between MDT care and improved local control is not solely attributable to measured baseline differences. By applying two complementary propensity score approaches—1:1 matching and stabilized inverse probability weighting—and observing concordant effect directions for 3-year local recurrence, we provide a consistent signal across analytic frameworks that make different assumptions and are susceptible to different forms of residual imbalance (23). In addition, reporting standardized mean differences alongside conventional hypothesis testing allows readers to judge covariate balance directly rather than relying on P values alone (24). Finally, presenting both fixed-time (3-year) endpoints and complementary time-to-event curves offers a clinically intuitive view of early relapse patterns, while preserving the primary focus on prespecified 3-year outcomes. This approach was considered appropriate in high-grade STS, where early local failure often reflects the quality of initial local therapy. Because the primary analyses were based on separate fixed 3-year endpoints rather than cumulative incidence of mutually exclusive first events, competing-risk modelling was not used in the present study; however, this may be a useful direction for future studies with more complete adjudication of competing events and longer follow-up.

Several limitations should be considered when interpreting these findings. First, the retrospective, non-randomized design remains vulnerable to residual confounding. Although propensity score methods were used, they can only adjust for measured variables; unmeasured factors, including detailed imaging features, molecular alterations, surgeon experience, margin width, radiotherapy target delineation and dose/fractionation, chemotherapy regimen, and time from diagnosis to treatment initiation, were not fully captured. Therefore, causality cannot be inferred from this observational study. Second, MDT exposure was defined according to documented pre-treatment discussion. Although MDT recommendations were recorded, adherence to individual recommendations was not systematically assessed, and informal or undocumented multidisciplinary input in the non-MDT group may have led to exposure misclassification. In addition, the quality or depth of MDT discussion could not be evaluated, and patients with more complex disease presentations may have been more likely to undergo formal MDT discussion, introducing the possibility of confounding by indication. Third, the study spanned 2010–2022, during which MDT practice, referral patterns, imaging quality, and treatment strategies may have evolved; therefore, potential time-period bias cannot be excluded. Fourth, FNCLCC grade 3 assignment was based on institutional pathology reports, and no additional central pathology re-review was performed specifically for this study. Finally, outcomes were ascertained from outpatient records and telephone follow-up, which may have introduced recall or ascertainment bias and affected the precision of event timing. As a single-center study from a national cancer center, our findings may not generalize to centers without formal sarcoma MDT infrastructure or high sarcoma case volume. Multicenter studies with standardized definitions, process-level MDT metrics, and longer follow-up are warranted.

From a clinical perspective, these results support embedding formal MDT review early in the care pathway for FNCLCC grade 3 STS, where the window for optimizing local therapy is narrow and the consequences of suboptimal initial management can be durable. In practice, our data suggest that the greatest value of MDT care may lie in its association with more consistent and higher-quality initial local treatment planning, including appropriate diagnostic work-up, coordinated surgical strategy, and rational integration of radiotherapy, which may in turn contribute to better local control. At the same time, the absence of a clear reduction in early distant metastasis highlights an unmet need for more effective systemic risk stratification and metastasis-directed strategies in high-grade STS. Future work should evaluate MDT “process” metrics (e.g., time to treatment initiation, adherence to MDT recommendations, margin quality, radiotherapy utilization and sequencing) and explore whether specific histologic subtypes or clinical presentations derive differential benefit from MDT care, ideally through multicenter collaborations with standardized endpoints and longer follow-up.

Overall, in a large single-center cohort of patients with FNCLCC grade 3 soft tissue sarcoma, pre-treatment MDT care was associated with a lower risk of 3-year local recurrence across multiple propensity score–based analytic approaches, whereas no statistically significant difference was observed for 3-year distant metastasis. These findings support the value of MDT-based management as a potential quality-improvement strategy for optimizing initial local therapy in high-grade STS. Further multicenter studies with standardized definitions, longer follow-up, and process-level measures of MDT implementation are warranted to clarify which patients benefit most and whether MDT care can influence late metastatic relapse when combined with optimized systemic strategies.

## Data Availability

The raw data supporting the conclusions of this article will be made available by the authors, without undue reservation.
