# Supplementary figures and images for: Multidisciplinary team care and outcomes in FNCLCC grade 3 soft tissue sarcoma: a propensity score–matched and weighted cohort study
Source: Front Oncol. 2026 Jun 10;16:1827237. doi: 10.3389/fonc.2026.1827237 (PMC13290705; doi:10.3389/fonc.2026.1827237)

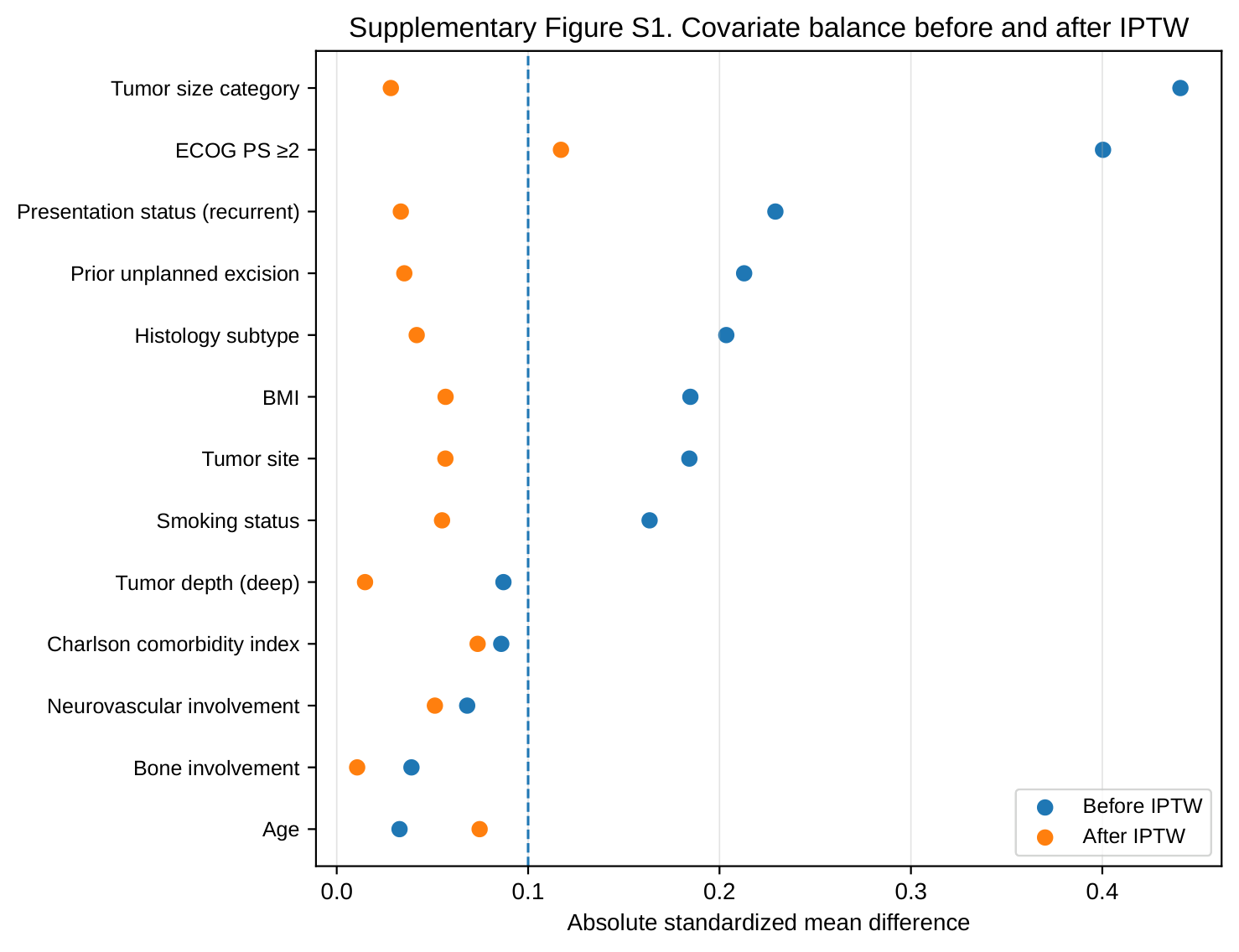

Supplement: Supplementary file 1 [file Image1.tiff]

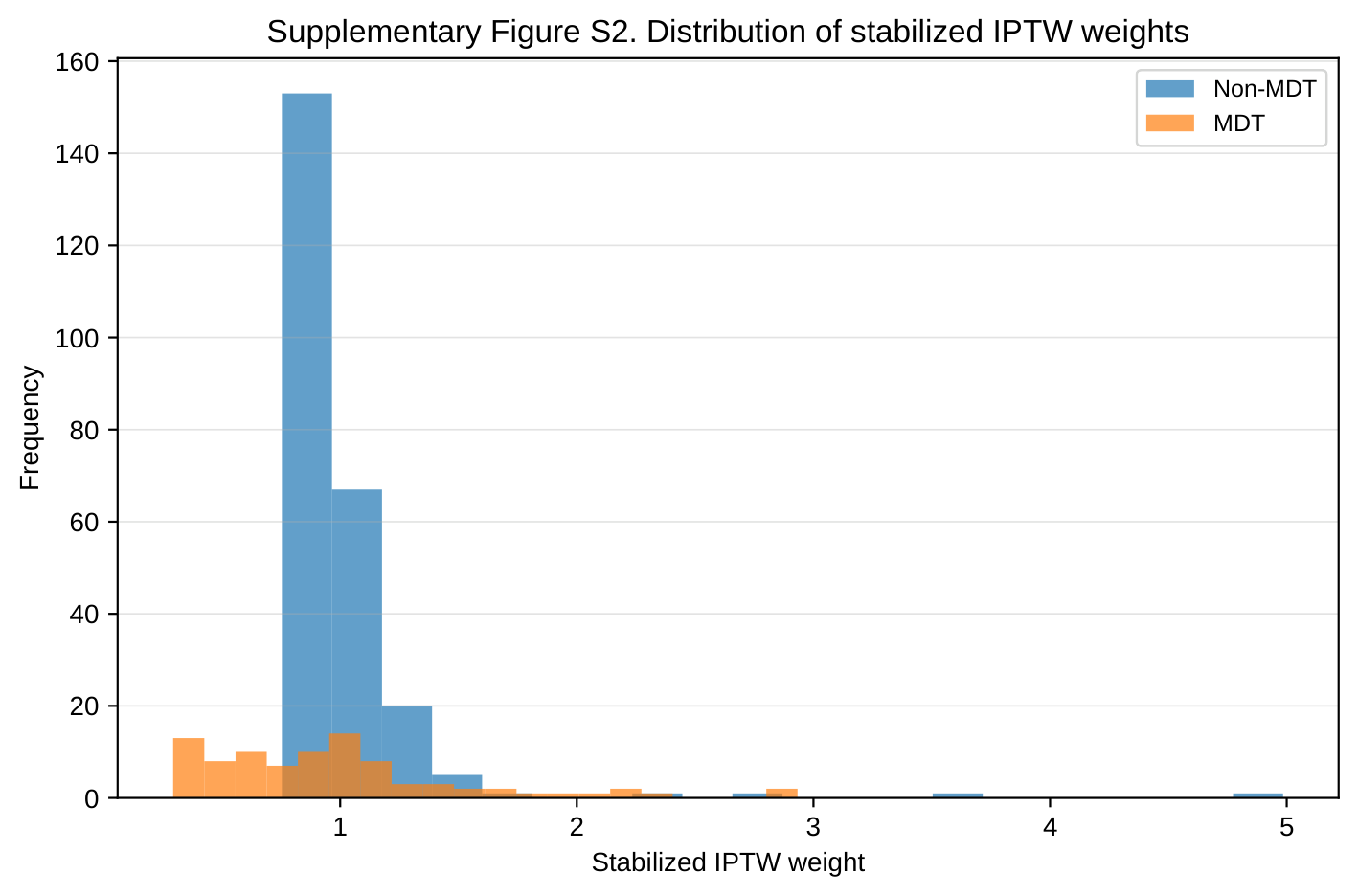

Supplement: Supplementary file 2 [file Image2.tiff]

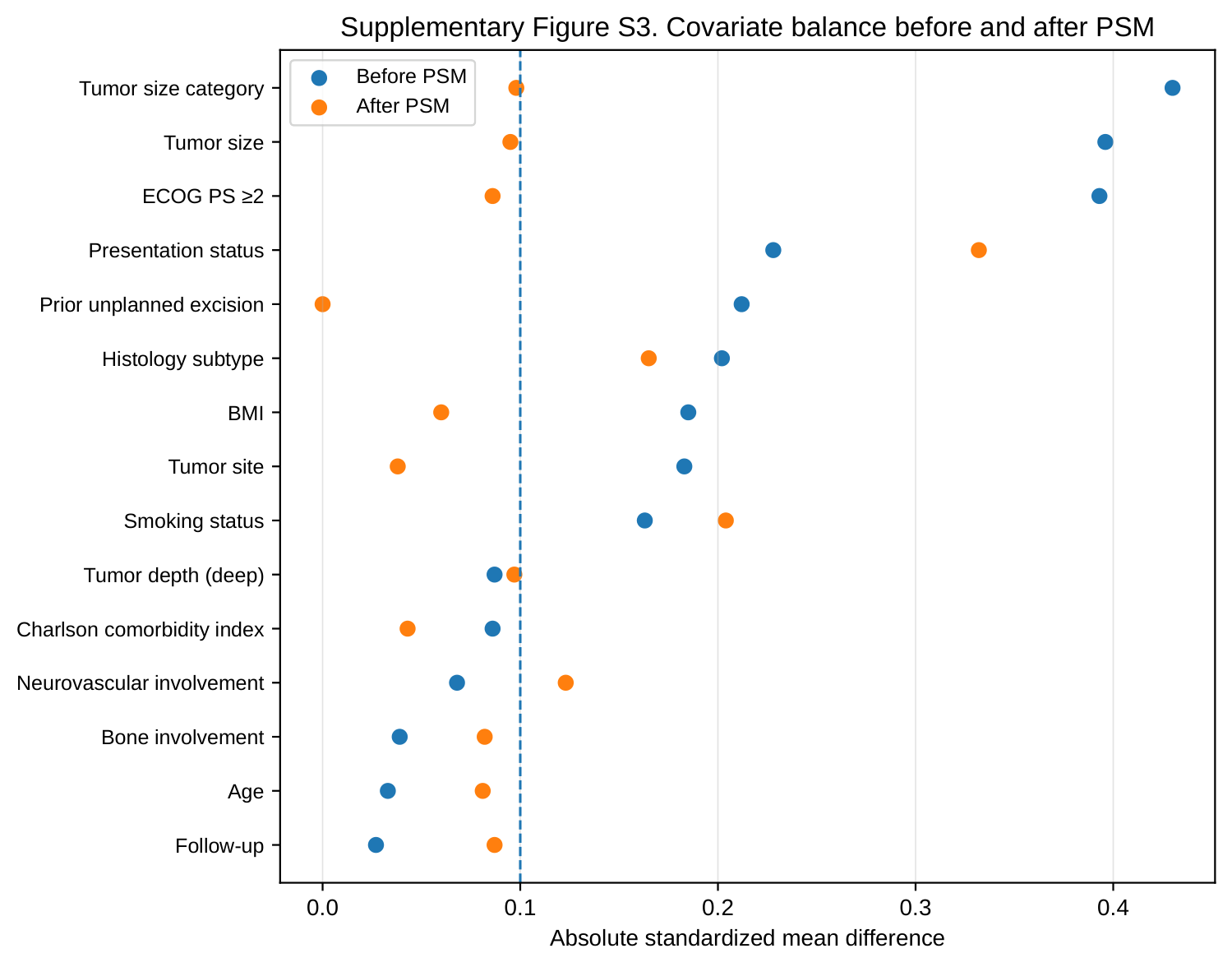

Supplement: Supplementary file 3 [file Image3.tiff]
